# Combined donor–recipient CYP3A functional allele count as an independent predictor of tacrolimus pharmacokinetics after liver transplantation

**DOI:** 10.3389/fphar.2026.1879383

**Published:** 2026-08-12

**Authors:** Luis Ramudo-Cela, Brais Muñiz-Castro, Francisco Suárez-López, Alejandra Otero-Ferreiro, Gilberto Pérez, José Santos, Pedro Cabalar, Luis Margusino-Framiñán

**Affiliations:** 1 Department of Hospital Pharmacy, Complexo Hospitalario Universitario de A Coruña (CHUAC), Sergas, A Coruña, Spain; 2 Hospital Pharmacy Research Group, Instituto de Investigación Biomédica de A Coruña (INIBIC), Complexo Hospitalario Universitario de A Coruña (CHUAC), Sergas, University of A Coruña (UDC), A Coruña, Spain; 3 Department of Computer Science and Information Technologies, University of A Coruña, A Coruña, Spain; 4 Research Center in Information and Communication Technologies (CITIC), University of A Coruña, A Coruña, Spain; 5 Department of Hepatology and Liver Transplantation, Complexo Hospitalario Universitario de A Coruña (CHUAC), Sergas, A Coruña, Spain

**Keywords:** Cytochrome P-450 CYP3A, drug metabolism, liver transplantation, pharmacogenetics, pharmacokinetics, precision medicine, tacrolimus, therapeutic drug monitoring

## Abstract

**Background:**

Tacrolimus (TAC) exhibits high pharmacokinetic variability post-liver transplantation (LT). Traditional models overlook the metabolic chimerism between recipient (intestinal) and donor (hepatic) genotypes. We aimed to evaluate a combined donor–recipient CYP3A functional allele count predicting TAC exposure.

**Methods:**

This retrospective study analyzed 1,350 TAC trough measurements from 99 LT recipients. *CYP3A4* and *CYP3A5* functional alleles were summed into a combined pair-level framework (0–8 alleles). The primary outcome was the log-transformed concentration-to-dose ratio (log-CDR). Linear mixed-effects models (LMM) accounted for repeated measurements, adjusting for clinical and pharmacological covariates.

**Results:**

The combined CYP3A functional allele count was the strongest genetic predictor of TAC exposure. Using the most prevalent group (4 alleles) as reference in multivariable LMMs, dose-normalized exposure significantly decreased by 43% and 48% in pairs with 5 and 6 functional alleles, respectively (p < 0.001). This gene-dose relationship remained stable across ICU, ward, and outpatient phases. The combined metric captured 4.2% of baseline variance (marginal *R* ^2^ = 0.042), indicating that most TAC exposure variability is driven by non-genetic clinical factors. Nevertheless, this approach doubled the explanatory power achieved with recipient genotype alone and remained an independent predictor after adjustment for major time-varying clinical confounders.

**Conclusion:**

The combined donor–recipient CYP3A functional allele count provides a biologically coherent, temporally stable metric of TAC metabolism. Outperforming single-genome models, it offers a stratification tool to guide early post-operative initial dose strategies.

## 1 Introduction

Tacrolimus (TAC) is the pharmacological cornerstone for the prevention of allograft rejection in solid organ transplantation (Henkel et al., 2023; Shao et al., 2020). However, TAC management is perpetually challenged by a narrow therapeutic index and substantial pharmacokinetic (PK) variability (Brunet et al., 2019). Standard bodyweight-based dosing frequently fails to achieve optimal target trough concentrations (C_0_) during the immediate post-operative window—the period of highest immunological vulnerability (Birdwell et al., 2015). Consequently, recipients face either subtherapeutic levels, risking acute cellular rejection, or supratherapeutic exposure, inducing nephrotoxicity, neurotoxicity, and post-transplant diabetes mellitus (Brunet et al., 2019; Brunet and Pastor-Anglada, 2022).

TAC systemic disposition is primarily governed by cytochrome P450 3A oxidative metabolism, specifically hepatic and intestinal *CYP3A4* and *CYP3A5* isoenzymes (Abuelsoud et al., 2024; Amaro-Álvarez et al., 2024; Sánchez and Montero, 2024). The CYP3A5*3 loss-of-function polymorphism is the most significant genetic determinant, where expressors (CYP3A5*1 carriers) exhibit significantly higher clearance, requiring 1.5- to 2-fold dose increases to achieve therapeutic targets (Birdwell et al., 2015; Du et al., 2024). In liver transplantation (LT), this landscape is further complicated by metabolic chimerism: intestinal first-pass metabolism is dictated by the recipient’s genotype, whereas systemic hepatic clearance depends on the donor’s genetic background (Nakamura et al., 2020).

Although Therapeutic Drug Monitoring (TDM) is the standard of care, it remains inherently retrospective (Brunet et al., 2019). Because single-genome analyses partially explain PK variance (Shao et al., 2020; Brunet et al., 2019), integrating *CYP3A4* and *CYP3A5* variants across the donor–recipient axis is necessary to improve predictive accuracy (Concha et al., 2024; Mulder et al., 2021; Hoffert et al., 2024).

Here, we aim to assess the combined pair-level *CYP3A* functional allele count as a predictor of the log-transformed concentration-to-dose ratio (log-CDR). By integrating the functional status of both *CYP3A4* and *CYP3A5* across the entire donor–recipient axis into a single quantitative metric, we hypothesized this biomarker would capture a higher proportion of PK variability than individual models. Given the inherent logistical challenges of obtaining donor genotypes during acute organ procurement, this metric is explicitly envisioned not as a pre-transplant initial dosing calculator. Instead, it is intended to function as an early post-operative stratification tool to guide subsequent progressive dose titrations once donor data is finalized, or as a framework for the retrospective clinical interpretation of extreme, unexplained TDM outliers, thereby providing a biologically integrated, time-invariant tool to guide post-operative TAC management in LT.

## 2 Materials and methods

### 2.1 Study design and population

This retrospective observational study included adult LT recipients (≥18 years) transplanted at a tertiary academic center between January 2019 and December 2021. Exclusion criteria comprised combined liver–kidney transplantation, retransplantation, and concurrent treatment with known moderate or strong inhibitors/inducers of *CYP3A4/5* (e.g., azole antifungals, macrolides, protease inhibitors, or rifampin) to prevent exogenous pharmacokinetic confounding and phenoconversion. The final cohort included 99 recipients contributing 1,350 post-transplant TAC trough (C_0_) observations. The institutional Ethics Committee approved the study (approval number 2021/287).

### 2.2 Data collection and pharmacokinetic outcomes

Demographic, clinical, and pharmacological variables were systematically captured using an electronic Case Report Form (Margusino-Framiñán et al., 2017). TAC C_0_ was measured via validated immunoassays (Abbott ARCHITECT, Abbott Diagnostics). All TAC trough concentrations (C_0_) were obtained from blood samples drawn strictly prior to the morning dose administration (approximately 12 h post-dose for twice-daily formulations and 24 h post-dose for once-daily formulations), adhering to standardized institutional TDM protocols. To account for the typical right-skewed distribution of TAC exposure, the primary pharmacokinetic outcome was the log-transformed concentration-to-dose ratio (log-CDR = log (C_0_/daily dose)). Patient location at the time of sampling (ICU, hospital ward, or home) was recorded as a proxy for the post-transplant clinical phase.

### 2.3 Genotyping and pharmacogenetic stratification

Genomic DNA was extracted from recipient and donor biobanked blood clots. Genotyping for CYP3A5*3 (rs776746) and CYP3A4*22 (rs35599367) variants was performed using TaqMan allelic discrimination assays (Applied Biosystems).

Following the Clinical Pharmacogenetics Implementation Consortium (CPIC) and Pharmacogene Variation Consortium (PharmVar) guidelines, alleles with normal enzymatic activity (CYP3A4*1, CYP3A5*1) were classified as functional and assigned 1 point (Gaedigk et al., 2019). Loss-of-function variants (CYP3A5*3, CYP3A4*22) were assigned 0 points (Concha et al., 2024). To model CYP3A-mediated metabolic capacity as a continuous gene–dose variable rather than relying on categorical classifications, functional alleles were summed. This yielded individual allele scores (0–4 for each donor and recipient) and our primary predictor: the combined donor–recipient *CYP3A* functional allele count (ranging from 0 to 8).

### 2.4 Statistical analysis

To appropriately account for the non-independence of repeated TAC measurements within patients, univariable, longitudinal, and multivariable analyses were conducted using linear mixed-effects models (LMM) with the patient identifier included as a random intercept. Univariable LMMs estimated fixed-effect β coefficients and relative exposure changes (exp(β)) for genetic predictors, establishing the most prevalent genotype group as the reference category.

Longitudinal LMMs evaluated the temporal stability of the genetic effect across evolving clinical phases (with “home” serving as the reference).

Multivariable analyses were executed using linear mixed-effects models (LMM) with the patient identifier included as a random intercept to account for longitudinal intra-individual correlation. To account for routine clinical switches between products over time within the same individual (e.g., transitioning from oral solutions or immediate-release formulations in the ICU to extended-release formulations in later phases), TAC formulation was modeled as a time-varying categorical covariate. The initial candidate variable pool entered into the statistical selection process comprised an exhaustive list of 34 parameters, including all baseline donor-recipient demographics (age, sex, BMI), pre-transplant biochemical parameters (AST, ALT, GGT, alkaline phosphatase, albumin, INR, total bilirubin), donor graft characteristics (type, steatosis), and time-varying post-transplant clinical, analytical, and pharmacological covariates (clinical phase, tacrolimus formulation, T-tube status, hematocrit, daily MMF, and corticosteroid doses). Multi-collinearity among candidates was verified utilizing Variance Inflation Factors (VIF), with all variables remaining well below the conservative threshold ( VIF<2.5 ). Bidirectional stepwise selection based on the Akaike Information Criterion (AIC) was applied to achieve model parsimony. To validate the structural stability of our model and address sample imbalances, two distinct sensitivity analyses were performed: (1) a forced-entry multivariable model incorporating all 34 initial candidate variables simultaneously without selection, and (2) a model treating the combined CYP3A functional allele count as a continuous linear predictor to characterize the overall gene-dose trajectory across the cohort. Furthermore, to formally evaluate the temporal stability of the genetic effect across evolving post-transplant stages, an interaction analysis was executed by introducing a fixed-effect interaction term between the combined CYP3A functional allele count and the clinical phase into the longitudinal LMM. The statistical significance of this interaction block was assessed utilizing a Type III Analysis of Variance (ANOVA) with Satterthwaite’s method for degrees of freedom calculation. Model explanatory power was quantified using marginal *R* ^2^ (variance explained by fixed factors) and conditional *R* ^2^ (variance explained by fixed and random factors). Analyses were performed using R version 4.3.3; statistical significance was defined as p < 0.05.

## 3 Results

### 3.1 Patient demographics and pharmacogenetic profile

Baseline demographic, clinical, and genetic characteristics of the 99 liver transplant recipients and their respective donors are summarized in Table 1. The cohort had a median recipient age of 60 years [IQR: 55–64] and a median pre-transplant MELD score of 10.0 [IQR: 6.5–14.0]. Reflecting the demographic composition of the geographical region during the study period, the cohort was entirely of white European descent (100% Caucasian).

**TABLE 1:** Baseline characteristics and summary of post-transplant follow-up observations.

Category	Variable	Unit/ Category	Summary
Baseline demographics	Recipient age/ Sex	years/ Male, n (%)	59.0 ± 7.4/ 75 (75.8%)
*(N = 99 pairs)*	Donor age/ Sex	years/ Male, n (%)	56.9 ± 14.3/ 57 (58.2%)
​	MELD score	Points, mean ± SD	11.2 ± 6.8
​	Pre-Tx diabetes/ HCC	Yes, n (%)	32 (32.3%)/ 44 (44.4%)
Pharmacogenetics	Recipient/ Donor *CYP3A5* status	0 functional alleles (non-expressors), n (%)	85 (85.9%)/ 81 (81.8%)
*(N = 99 patients)*	Recipient/ Donor *CYP3A4* status	2 functional alleles, n (%)	93 (93.9%)/ 93 (93.9%)
​	Combined pair count	3/ 4/ 5/ 6 alleles, n (%)	9 (9.1%)/ 65 (65.7%)/ 19 (19.2%)/ 6 (6.1%)
Follow-up summary	Clinical phase	Home/ Ward/ ICU, n (%)	588 (43.6%)/ 375 (27.8%)/ 387 (28.7%)
*(N = 1,350 observations)*	TAC formulation	Envarsus®/ Advagraf®/ Prograf®/ Oral, n (%)	479 (35.5%)/ 474 (35.1%)/ 397 (29.4%)/ 14 (1.0%)
​	T-tube status	Clamped, n (%)	819 (60.7%)
Analytical and PK	Hematocrit	%, mean ± SD	33.3 ± 6.2
​	TAC trough (C_0_)	ng/mL, mean ± SD	8.05 ± 4.61
​	TAC CDR	ng/mL/mg, mean ± SD	1.53 ± 1.40
​	Log-transformed CDR	Log-unit, mean ± SD	0.07 ± 0.88

Data are presented as mean ± standard deviation or frequency (percentage). Baseline demographics and donor-recipient genetic profiles correspond to the cohort of 99 transplant pairs (N = 99). Follow-up context, analytical markers, and pharmacokinetic outcomes reflect the summary of 1,350 longitudinal observations collected over a mean follow-up of 8 months. Abbreviations: CDR, concentration-to-dose ratio (C0/ daily dose); HCC, hepatocellular carcinoma; ICU, intensive care unit; MELD, Model for End-Stage Liver Disease; TAC, tacrolimus. Combined *CYP3A* functional allele count includes the sum of *CYP3A4* and *CYP3A5* functional alleles from both donor and recipient. The 4-allele group was established as the statistical reference category based on its prevalence in the study population. Log-CDR, values are derived from the total pool of longitudinal samples. Raw CDR, and Log-transformed CDR, are presented simultaneously to clarify the mathematical distribution used for clinical modeling.

Consequently, the pharmacogenetic profile showed a high prevalence of *CYP3A5* non-expressers in both recipients (85.9%) and donors (81.8%). Conversely, the *CYP3A4* genotype was highly conserved, with 93.9% of recipients and donors carrying two functional alleles. For our primary analytical variable—the combined total number of functional *CYP3A* alleles in the donor–recipient pair—the distribution was as follows: 9.1% of pairs had 3 alleles, 65.6% had 4 alleles, 19.2% had 5 alleles, and 6.1% had 6 alleles. No pair presented with fewer than 3 functional alleles.

### 3.2 Longitudinal clinical and pharmacokinetic data

The longitudinal analysis incorporated 1,350 post-transplant TAC observations, collected over a mean follow-up period of 8 months. These measurements captured the full continuum of post-operative recovery: 28.7% were obtained while patients were in the intensive care unit (ICU), 27.8% in the digestive hospital ward, and 43.5% after discharge in stable home settings.

Regarding immunosuppressive therapy, the most frequently prescribed TAC formulations were prolonged-release tablets (Envarsus®, 35.5%; Advagraf®, 35.1%), followed by immediate-release capsules (Prograf®, 28.3%). The mean TAC daily dose across all observations was 7.49 ± 4.34 mg/day, resulting in a mean C_0_ of 8.05 ± 4.61 ng/mL and a mean CDR of 1.53 ± 1.40 ng/mL/mg. Concomitant immunosuppression was standard, including mycophenolate mofetil (mean 1315.6 ± 636.8 mg/day) and tapering steroids (Supplementary Table S1).

### 3.3 Univariate pharmacogenetic analysis

**FIGURE 1:**
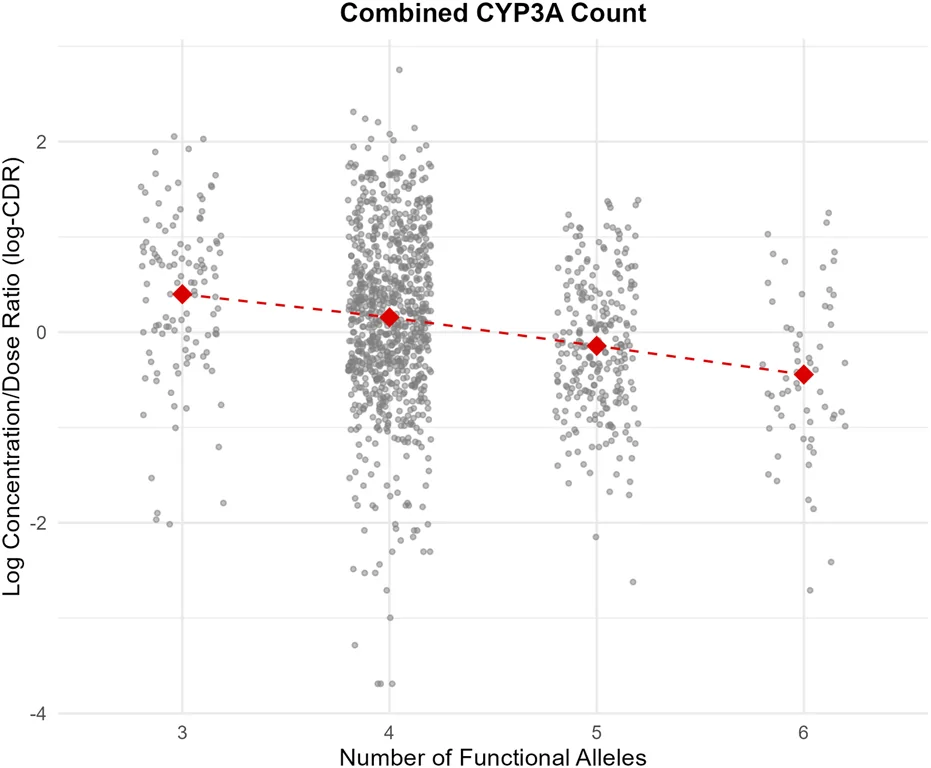
Dotplots of log-CDR stratified by combined donor–recipient CYP3A functional allele count. Individual data points represent the raw observed log-CDR values for tacrolimus measurements stratified by the combined donor–recipient CYP3A functional allele count. The red points indicate the unadjusted mean log-CDR for each genotype group.

When results were expressed as relative changes in exposure using exponentiated coefficients (exp(β)), a clear gene–dose effect was observed (Table 2; Figure 1). The combined donor–recipient *CYP3A* functional allele count demonstrated a strong, graduated association with TAC exposure. Compared with the 4-allele reference group, dose-normalized exposure significantly decreased by 24% in pairs carrying 5 functional alleles (β = −0.27, 95% CI –0.52 to −0.03; p = 0.014) and by 44% in pairs with 6 functional alleles (β = −0.58, 95% CI –0.99 to −0.17; p = 0.003). Conversely, an exploratory assessment of pairs carrying only 3 functional alleles exhibited a 27% increase in dose-normalized concentrations (β = 0.24, 95% CI –0.10–0.58), though this trend did not reach strict statistical significance ( p=0.085 ), likely due to the smaller sample size of this extreme stratum ( n=9 ). Given the small and imbalanced sample sizes within the extreme 3-allele ( n=9 ) and 6-allele ( n=6 ) groups, their specific confidence intervals in stratified models must be interpreted with caution. However, the pre-planned sensitivity analysis modeling the allele count as a continuous linear predictor confirmed a highly significant, progressive negative metabolic trend across the entire genetic continuum ( β=−0.394 per additional functional allele, SE=0.029 , t=−13.51 , $p=7.75\times 10^{-38}$ ). This linear trend demonstrates that each additional functional allele contributed by the donor-recipient pair significantly and progressively reduces dose-normalized tacrolimus exposure, confirming a stable gene-dose biological gradient that is not driven by extreme strata artifacts.

**TABLE 2:** Univariable Association Between Genetic Predictors and Tacrolimus log-CDR.

Genetic predictor	Category	N (pairs)	β coefficient	exp(β)	Relative change	95% CI (β)	p-value
Combined CYP3A count	4 alleles (ref)	65	—	1.00	Reference	—	—
​	3 alleles	9	+0.24	1.27	+27%	−0.10 to 0.58	0.085
​	5 alleles	19	−0.27	0.76	−24%	−0.52 to −0.03	0.014
​	6 alleles	6	−0.58	0.56	−44%	−0.99 to −0.17	0.003
Recipient *CYP3A5*	0 alleles (ref)	85	—	1.00	Reference	—	—
​	1 allele	14	−0.31	0.73	−27%	−0.60 to −0.02	0.017
Donor *CYP3A5*	0 alleles (ref)	81	—	1.00	Reference	—	—
​	1 allele	18	−0.43	0.65	−35%	−0.69 to −0.18	<0.001

Linear mixed-effects models (LMM) were fitted using the patient identifier as a random intercept to account for the non-independence of repeated observations (N = 1,271). The β coefficients represent the fixed-effect estimate of the change in the log-transformed concentration-to-dose ratio (log-CDR). Exp(β) represents the ratio of the geometric means, reflecting the relative change in dose-normalized exposure compared to the reference category. Abbreviations: CI, confidence interval; exp(β), exponentiated coefficient; ref, reference category. For the combined donor-recipient count, the 4-allele group was selected as the reference based on its highest prevalence. For individual genes, the reference represents the homozygous functional or non-expressor genotype as indicated. All models were adjusted for intra-patient correlation.

To quantify the explanatory power of these genetic models within the complex nature of TAC disposition, we assessed both marginal and conditional *R* ^2^ metrics. As expected in the highly dynamic early post-transplant setting, intrinsic intra-patient variability accounted for a large portion of the overall variance, with the full mixed-effects models capturing 27.6% of the total variability (conditional *R* ^2^ = 0.276). However, when isolating the specific predictive power of the baseline genetics (marginal *R* ^2^), the integrated pair-level metric proved clearly superior to single-genome models. While individual recipient and donor *CYP3A* genotypes explained only a modest 2.1% and 3.4% of the variance, respectively, the combined donor–recipient *CYP3A* functional allele count essentially doubled the baseline explanatory capacity of the recipient’s genotype, capturing 4.2% of the variance (marginal *R* ^2^ = 0.042) (Supplementary Figure S1; Supplementary Table S2). However, it is critical to note that the marginal explanatory power of this univariable genetic model is modest, establishing that while genetics provides a significant baseline metabolic floor, approximately 95.8% of the fixed-effect pharmacokinetic variance remains unexplained or is modulated by dynamic clinical noise.

### 3.4 Longitudinal analysis across post-transplant phases

**FIGURE 2:**
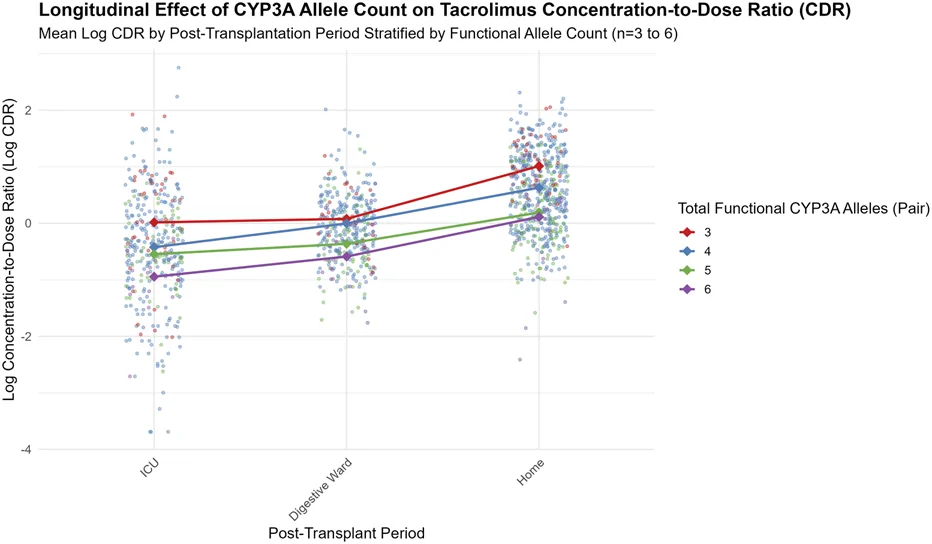
Longitudinal trajectories of log-CDR across post-transplant phases Mean log-CDR values across post-transplant clinical phases (home, digestive ward, ICU), stratified by combined CYP3A functional allele count (three, four, five, or six alleles). Data points represent the patient-lever raw values, and the lines represent the unadjusted means.

In longitudinal mixed-effects analyses stratified by the combined functional allele count, the genetic variable showed a consistent effect across all post-transplant clinical phases (Figure 2; Supplementary Table S3). A systematic reduction in absolute TAC exposure was observed during hospitalization compared with the home setting across all genetic strata. Specifically, both digestive ward admission and ICU stays were associated with significantly lower log-CDR values relative to the home phase (reductions ranging from 41% to 66%, all p < 0.001). To confirm this temporal stability mathematically, a formal interaction analysis was conducted by introducing an interaction term between the genetic strata and the clinical phase into the LMM. The Type III Analysis of Variance confirmed a non-significant interaction ( F=1.739,p=0.109 ), formally demonstrating that the predictive effect of the combined genetic metric remains statistically stable and independent of the post-transplant recovery phase. Importantly, despite these massive clinical shifts, the relative ordering of exposure between the genetic strata remained strictly preserved throughout the follow-up.

### 3.5 Multivariable analysis

**FIGURE 3:**
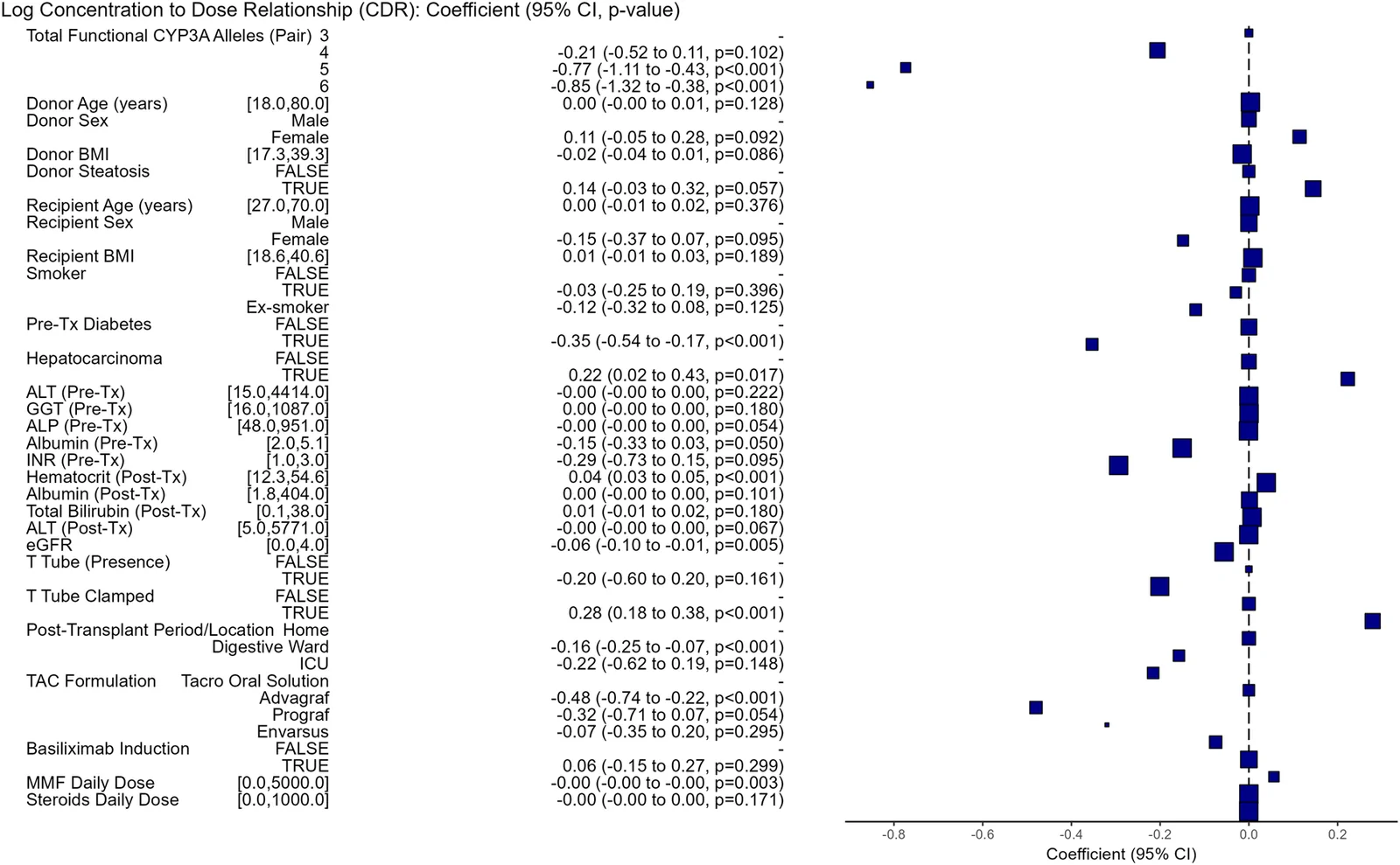
Multivariable linear regression of log-transformed TAC log-CDR. Forest plot comparing β coefficients and 95% confidence intervals for major *CYP3A* genetic predictors across univariable and multivariable linear regression models. The effect sizes reflect the changes in log-CDR relative to each reference genotype. Plotted values represent model-adjusted estimates ( β -coefficients) and their corresponding 95% confidence intervals derived from the final multivariable LMM.

In the final multivariable LMM, which adjusted for demographic, clinical, biochemical, and pharmacological covariates, the combined donor–recipient *CYP3A* functional allele count remained an independent and potent predictor of TAC exposure (Figure 3). The final multivariable model included 1,271 observations; 79 observations (5.8% of the total dataset) were excluded from this specific model via listwise deletion due to missing concurrent data in one or more of the adjusted dynamic clinical covariates (e.g., missing same-day hematocrit or biochemical markers).

Using the 4-allele cohort as the reference, the model confirmed the allele count–dependent reduction in TAC exposure: while patients carrying 3 functional alleles showed a non-significant trend toward higher dose-normalized exposure ( β=0.21 , 95% CI –0.11 to 0.52; p=0.102 ), patients carrying 5 functional alleles showed an estimated 43% reduction in dose-normalized exposure ( β=−0.57 , 95% CI –0.79 to −0.35; p<0.001 ), while those with 6 functional alleles demonstrated a 48% lower exposure ( β=−0.65 , 95% CI –1.02 to −0.28; p<0.001 ).

By controlling for intra-individual correlation, the mixed-effects model refined the adjusted impact of clinical covariates. Post-transplant hematocrit emerged as a highly significant dynamic determinant, with increasing values driving higher whole-blood TAC exposure (β = 0.04, p < 0.001). T-tube clamping was also independently associated with a significant increase in dose-normalized concentrations (β = 0.28, p < 0.001), underscoring the role of biliary excretion. TAC formulation significantly modulated exposure; compared to TAC oral solution, the Advagraf® formulation was associated with significantly lower dose-normalized exposure (β = −0.48, p < 0.001). Finally, pre-transplant diabetes mellitus (β = −0.35, p < 0.001) and estimated glomerular filtration rate (eGFR) remained independent predictors of pharmacokinetic variability, while markers of early graft function (such as bilirubin) lost independent significance.

## 4 Discussion

The primary objective of this study was to evaluate the combined donor–recipient *CYP3A* functional allele count as an independent predictor of TAC exposure during the early and clinically critical phase following LT. By controlling for the non-independence of repeated intra-patient sampling using LMM, we successfully isolated the genetic and physiological drivers of TAC disposition from statistical artifacts common in standard linear regressions.

Our findings demonstrate that this integrated pharmacogenetic metric explains a modest but independent proportion of inter-individual PK variability (marginal $R^{2}=4.2\%$ ), transparently underscoring that most of the TAC pharmacokinetic variability remains unexplained by the allele-count metric alone and is heavily driven by dynamic clinical noise. Nevertheless, it provides a biologically coherent baseline framework for dose optimization support in a setting characterized by a narrow therapeutic index and marked PK instability (Hoffert et al., 2024). The sample size of our cohort (n = 99) falls within the typical range for pharmacogenetic investigations in LT, which generally include between 50 and 200 patients. While larger multicenter cohorts or smaller, specialized studies exist, our population size provides sufficient statistical power to evaluate the gene–dose effect of the combined donor–recipient metric while maintaining high granularity in longitudinal clinical data.

Consistent with previous reports, we observed pronounced variability in the CDR, particularly during the early post-transplant period, where conventional weight-based dosing frequently fails to achieve timely therapeutic exposure (Henkel et al., 2023; Lu et al., 2021). This variability reflects the unique metabolic context of LT, in which drug disposition evolves dynamically as metabolic control transitions from recipient intestinal metabolism to donor hepatic metabolism (Du et al., 2024).

### 4.1 TAC pharmacogenetics: beyond single-gene models

Although *CYP3A5* is a major determinant of TAC clearance (Birdwell et al., 2015; Brunet and Pastor-Anglada, 2022), our results confirm that *CYP3A4* variants provide critical complementary information, especially in the context of liver transplantation chimerism (Brunet et al., 2019; Mulder et al., 2021).

In the context of LT, however, systemic exposure is governed by the combined metabolic activity of the donor liver and the recipient intestine, creating a chimeric pharmacokinetic system (Nakamura et al., 2020; Berger et al., 2019). Prior studies evaluating donor and recipient genotypes independently have yielded partially conflicting results regarding their relative temporal dominance. Recipient *CYP3A5* genotype appears to be more influential in the immediate post-operative period, whereas donor genotype gains relevance as graft function recovers (Nakamura et al., 2020; Liu et al., 2018; Naushad et al., 2019).

Quantitatively, integrated donor–recipient analyses have demonstrated clinically relevant effects, including a 1.7-fold increase in clearance when both donor and recipient carry functional *CYP3A5* alleles (Shao et al., 2020) and substantial differences in dose-adjusted exposure during the first month post-transplant (Nakamura et al., 2020; Naushad et al., 2019; Pinon et al., 2021). However, these approaches have largely relied on categorical comparisons of individual genes, limiting their ability to reflect cumulative metabolic capacity.

### 4.2 Allele-count integration as a unifying pharmacogenetic framework

Our findings extend this literature by demonstrating that a single quantitative metric—the combined donor–recipient *CYP3A* functional allele count—can summarize the total metabolic burden in a stable and biologically meaningful manner. The observed gene–dose relationship, with up to a 57% reduction in exposure between extreme allele categories, is comparable to or greater than effects reported for isolated donor or recipient genotypes.

This approach aligns with emerging allele-count and composite phenotype strategies. Brunet et al. (2019) and Woillard et al. (2017) demonstrated that combined *CYP3A4*/*CYP3A5* metabolizer clusters explain over 60% of variability in dose-adjusted concentrations, with extensive metabolizers requiring nearly threefold higher starting doses than poor metabolizers (Brunet et al., 2019; Woillard et al., 2017). Our work expands this paradigm by incorporating both donor and recipient genetics and preserving a continuous gene–dose relationship that directly reflects transplant-specific physiology.

Further support for cumulative genetic effects is provided by Coller et al. (2019), who reported progressively lower dose-adjusted concentrations with increasing combined donor–recipient *CYP3A* expression (Coller et al., 2019). Unlike prior trend-based analyses, our study formalizes this concept into a reproducible allele-count metric suitable for clinical modeling and decision support. Importantly, the structural integrity of this biological gradient was mathematically confirmed through a continuous linear trend sensitivity analysis, demonstrating a highly significant and progressive decline of −0.394 log-CDR units per additional functional allele ( $p=7.75\times 10^{-38}$ ), proving that the gene-dose trajectory remains highly stable despite the smaller sample sizes in the extreme strata.

### 4.3 Temporal independency across post-transplant phases

A key strength of the combined *CYP3A* functional allele count is its temporal stability across the highly dynamic post-transplant period. TAC pharmacokinetics undergo marked fluctuations during the transition from the acute postoperative phase to long-term maintenance, driven by graft recovery, corticosteroid exposure, and changes in clinical setting (Henkel et al., 2023). In the early post-transplant period (POD 1–28), high-dose corticosteroids induce *CYP3A4* activity, increasing TAC clearance and necessitating higher weight-adjusted doses; as steroids are tapered, clearance decreases and dose-adjusted concentrations rise (Brunet et al., 2019; Abuelsoud et al., 2024; Lu et al., 2021). In parallel, the relative contribution of genetic determinants evolves over time, with recipient intestinal *CYP3A5* activity predominating in the earliest phase and donor hepatic metabolism becoming increasingly influential as graft function stabilizes (Nakamura et al., 2020; Liu et al., 2018).

Despite these substantial shifts in absolute exposure, our longitudinal analysis shows that the relative ordering of TAC exposure across combined genetic strata remains preserved from the intensive care unit through hospital discharge and outpatient follow-up. This contrasts with single-genotype models—either donor- or recipient-based—whose predictive relevance may vary according to the phase of recovery.

### 4.4 Independence from clinical and biochemical confounders

To minimize potential residual confounding inherent to real-world longitudinal data, our multivariable LMM actively adjusted for the comprehensive matrix of time-varying physiological and clinical shifts that dictate tacrolimus disposition. Fluctuations in graft function were controlled via longitudinal transaminases and bilirubin tracking, while dynamic metabolic enzyme induction was accounted for by entering daily corticosteroid doses as a continuous time-varying factor. Within-patient formulation switching was natively handled by the mixed model as a time-varying categorical predictor mapped directly to the exact day of each sampling event. Crucially, potential feedback loops stemming from subsequent TDM-guided clinical dose adjustments were bypassed by utilizing the dose-normalized concentration-to-dose ratio (log-CDR) as our primary outcome variable, thereby isolating changes in intrinsic clearance from human-driven dosing decisions.

The independence of the combined genetic effect was confirmed in a fully adjusted multivariable model incorporating demographic, biochemical, physiological, and pharmacological covariates known to influence TAC pharmacokinetics. Consistent with prior studies, longitudinal changes in hematocrit emerged as a key determinant of whole-blood exposure, reflecting the extensive sequestration of TAC within erythrocytes and the risk of exposure misclassification in anemic patients early after transplantation (Henkel et al., 2023; Abuelsoud et al., 2024; Li et al., 2023; Piletta-Zanin et al., 2021). Likewise, bile drainage and T-tube clamping status were independently associated with a significant increase in exposure, reinforcing the central role of biliary excretion and enterohepatic recirculation in TAC clearance (Henkel et al., 2023; Shao et al., 2020; Abuelsoud et al., 2024; Hoffert et al., 2024).

Pharmacological factors further modulated TAC disposition independently of the genetic background. TAC formulation significantly influenced exposure, with specific extended-release preparations like Advagraf® exhibiting different bioavailability profiles compared to oral solutions (p < 0.001) (Henkel et al., 2023; Martial et al., 2021). Regarding demographic and clinical variables, pre-transplant morbidities such as diabetes mellitus and hepatocellular carcinoma maintained a strong independent association with altered drug exposure (Henkel et al., 2023; Nakamura et al., 2020).

Importantly, we must acknowledge that in observational retrospective cohorts, the risk of residual confounding—such as unmeasured drug-drug interactions, patient compliance outside the hospital, or subclinical gastrointestinal motility changes—can never be completely eliminated. However, the fact that rigorous statistical adjustment for this comprehensive matrix of dynamic variables (specifically addressing early graft function recovery, steroid exposure induction, formulation switching, TDM-driven adjustments, hematocrit changes, and bile drainage/T-tube status) did not attenuate the effect size of the higher combined allele counts (5 and 6 alleles) strongly indicates that the metric reflects an intrinsic biological gradient. Furthermore, by modeling the clinical phase location proxies (ICU, hospital ward, or home) as longitudinal covariates that mirror physiological recovery relative to the time after transplantation, we confirmed that these genetic strata maintained their relative predictive ordering across the entire post-transplant timeline. This stable stratification demonstrates a remarkable resilience against the temporal clinical noise that typically confounds single-genome models.

### 4.5 Limitations and future directions

This study has several important limitations. First, it was a single-center study conducted in a relatively small (n = 99) and predominantly Caucasian population. Consequently, the prevalence of *CYP3A5* expressors was low, with only 14 recipients and 18 donors carrying functional *CYP3A5* alleles. This limits the generalizability of the absolute effect sizes to populations with different allele frequencies. This demographic constraint underscores the need for future large-scale, multicenter studies in diverse populations to fully validate our findings. Nevertheless, the allele-count framework itself is inherently transferable and may provide even greater discrimination in genetically diverse cohorts where the dynamic range of the combined allele count might be more pronounced.

Second, from a logistical standpoint, we acknowledge that while pre-transplant genotyping of the recipient is highly feasible, obtaining the donor’s CYP3A genotype prior to graft reperfusion remains a practical challenge in the context of deceased donor liver transplantation. To clarify its clinical implementation pathway, this metric is explicitly not proposed as a pre-transplant initial dosing selector. Instead, it is intended to function as an early post-operative stratification tool—applied within the first week post-transplant once donor genetic data is finalized—to guide progressive dose titration. Furthermore, it serves as a tool for retrospective pharmacokinetic interpretation, enabling clinicians to identify the underlying biological causes of extreme, unexplained TDM outliers. Its utility as a true pre-transplant predictor will depend on the broader implementation of rapid, point-of-care genotyping technologies in donor evaluation protocols.

Third, a specific limitation of our proposed model is the assignment of equal functional capacity to all alleles within the count. While this simplification provides a practical and straightforward tool for clinical calculation at the point of care, it may not perfectly reflect potential nuances in the varying catalytic efficiencies of different isoenzymes or specific variants. Furthermore, our parsimonious model focused exclusively on the primary CPIC-recognized signals (CYP3A5*3 and CYP3A4*22). We did not include variants such as CYP3A4*1B (due to its strong linkage disequilibrium with CYP3A5*1 in Caucasians), nor secondary modulators like POR*28 or *NR1I2*. While these variants possess growing evidence of influence on early TAC clearance, their incorporation into a dual donor-recipient matrix would require significantly larger cohorts to avoid statistical overfitting. Evaluating whether these secondary modifiers add incremental predictive value to our CYP3A-centered framework remains a critical future direction. Although TAC pharmacokinetics are influenced by multiple genetic pathways beyond *CYP3A4* and *CYP3A5*, most additional loci described in the literature exert effects that are modest or context-dependent when compared with the dominant CYP3A signal. In particular, common *ABCB1* variants have not shown a consistent association with systemic TAC concentrations in LT (Naushad et al., 2019). Other metabolic regulators and transporter genes have been linked to specific phenotypes, but their contributions are generally secondary and often insufficient to meaningfully improve dose prediction beyond CYP3A-based models (Brunet and Pastor-Anglada, 2022; Abuelsoud et al., 2024; Nakamura et al., 2020; Coller et al., 2019; Chen et al., 2014; Ladd et al., 2025; Sendra et al., 2022).

Finally, we must acknowledge that while the combined allele count captures a significant portion of PK variance, substantial inter-individual variability remains even within a single functional allele group, as illustrated by the dispersion in our cohort. Prompted by these considerations, an exploratory analysis was conducted to correlate our genetic metric with hard clinical outcomes at the patient level (N = 99 pairs). No statistically significant associations were observed for acute cellular rejection (p = 0.78), acute kidney injury (p = 0.31), or post-transplant infections (p = 0.56). This lack of significance is expected due to the low baseline incidence of these events, which renders the study underpowered when clinical endpoints are distributed across multiple genetic strata. Previous prospective trials evaluating genotype-based tacrolimus dosing have yielded mixed or disappointing clinical results, underscoring that genetics alone cannot overcome the totality of PK instability. Crucially, the proposed genetic score is explicitly intended to complement, rather than replace, routine therapeutic drug monitoring (TDM) in clinical practice. Since this study lacks external replication and prospective validation, we cannot claim that this framework improves target attainment, reduces toxicity, or prevents rejection. Therefore, the clinical translation of this metric remains exploratory. Our model should not be viewed as a definitive solution or a standalone dosing calculator, but rather as a consistent, complementary baseline metric that, when combined with subsequent longitudinal TDM and clinical judgment, can help mitigate extreme outliers in early post-transplant TAC exposure. Future work should move toward the development of integral models, validated in prospective trials, that transcend genetic variables by incorporating a wider array of dynamic clinical and physiological factors. In this context, artificial intelligence and machine learning techniques may be particularly useful for processing and synthesizing these complex, multidimensional datasets to enhance the accuracy of personalized dosing algorithms.

## 5 Conclusion

In conclusion, this study evaluates the combined donor–recipient CYP3A functional allele count as an independent predictor of TAC pharmacokinetics, demonstrating a consistent gene–dose relationship across post-transplant clinical phases. By integrating hepatic and intestinal metabolic contributions into a single quantitative metric, this variable offers a more comprehensive assessment of baseline metabolic capacity than individual donor or recipient genotypes alone. While rapid donor genotyping presents logistical challenges, and substantial inter-patient variability inevitably remains, incorporating this biologically grounded assessment into early post-operative dosing strategies provides a promising, complementary tool to support early post-operative dosing strategies. Prospective, randomized trials are warranted to determine whether this integrated approach translates into improved clinical outcomes and reduced toxicity compared to standard therapeutic drug monitoring.

## Data Availability

The datasets presented in this article are not readily available in public repositories due to European and national legal and ethical restrictions regarding sensitive patient genomic and transplant information. Requests to access the datasets should be directed to the corresponding author.
