# Supplementary material for: Combined donor–recipient CYP3A functional allele count as an independent predictor of tacrolimus pharmacokinetics after liver transplantation
Source: Front Pharmacol. 2026 Aug 12;17:1879383. doi: 10.3389/fphar.2026.1879383 (PMC13506904; doi:10.3389/fphar.2026.1879383)
Supplement: Supplementary file 1 [file Supplementaryfile1.docx]

Supplementary Material

# 1 Supplementary Data

# 2 Supplementary Figures and Tables

## 2.1 Supplementary Figures

**Supplementary Figure 1.** Dotplots of log-CDR stratified by genetic predictors.

Distribution of log-transformed concentration/dose ratio (log-CDR) across genotype categories for key pharmacogenetic predictors. Panels include: (A) combined CYP3A count, (B) recipient CYP3A5, (C) donor CYP3A5, (D) recipient CYP3A4, (E) donor CYP3A4, (F) recipient combined CYP3A functional allele count, (G) donor combined CYP3A functional allele count, (H) combined donor–recipient CYP3A4 functional allele count, and (I) combined donor–recipient CYP3A5 functional allele count. Red points indicate the mean log-CDR for each genotype group.

## 2.2 Supplementary Tables

**Supplementary Table 1. Additional Pre- and Post-Transplant Parameters and Donor Characteristics.**

| Category | Variable | Category / Unit | Summary |
| --- | --- | --- | --- |
| Recipient/Donor Details | Recipient BMI | kg/m², mean ± SD | 27.7 ± 4.7 |
|  | Donor BMI | kg/m², mean ± SD | 26.1 ± 3.9 |
|  | Donor Type | DBD / DCD, n (%) | 62 (62.6%) / 37 (37.4%) |
|  | Donor Steatosis | Present, n (%) | 62 (62.6%) |
|  | Smoking Status | Never / Former / Current, n (%) | 43 (43.4%) / 27 (27.3%) / 29 (29.3%) |
| Pre-Tx Biochemistry | AST / ALT | U/L, mean ± SD | 258.8 ± 666.1 / 243.9 ± 659.9 |
| (N=99) | Albumin / INR | g/dL / ratio, mean ± SD | 3.52 ± 0.69 / 1.47 ± 0.29 |
|  | Total Bilirubin | mg/dL, mean ± SD | 3.06 ± 4.75 |
| Follow-up Parameters | AST / ALT | U/L, mean ± SD | 98.8 ± 299.8 / 204.8 ± 401.8 |
| (N=1,350) | Total Bilirubin | mg/dL, mean ± SD | 1.77 ± 2.57 |
|  | Estimated GFR | points (0–4), mean ± SD | 3.24 ± 0.89 |
| Co-medications | MMF Dose | mg/day, mean ± SD | 1315.6 ± 636.8 |
|  | Steroid Dose | mg/day, mean ± SD | 33.3 ± 61.5 |

Data are presented as mean ± standard deviation or frequency (percentage). Baseline biochemistry (N=99) corresponds to the last available assessment prior to graft reperfusion. Post-transplant biochemical and hematological parameters (N=1,350) represent the cumulative summary of all measurements during the follow-up period. **Abbreviations:** ALT, alanine aminotransferase; AST, aspartate aminotransferase; BMI, body mass index; DBD, donation after brain death; DCD, donation after circulatory death; GFR, glomerular filtration rate; INR, international normalized ratio; MMF, mycophenolate mofetil. **Notes:** Dosing for mycophenolate mofetil and steroids (prednisone-equivalent) represent the average daily maintenance dose across the 1,350 observations. Estimated GFR was categorized according to clinical ranges (0–4) consistent with institutional protocols.

**Supplementary Table 2. Additional univariable linear mixed-effects models for secondary individual and pair-level genetic predictors.**

| Genetic predictor | Category | n (pairs) | β coefficient | exp(β) | Relative change | 95% CI (β) | p-value |
| --- | --- | --- | --- | --- | --- | --- | --- |
| Recipient CYP3A4 alleles | 1 (ref) | 6 | — | 1.00 | Reference | — | — |
|  | 2 alleles | 93 | –0.23 | 0.79 | –21% | –0.64 to 0.18 | 0.132 |
| Donor CYP3A4 alleles | 1 (ref) | 6 | — | 1.00 | Reference | — | — |
|  | 2 alleles | 93 | –0.02 | 0.98 | –2% | –0.44 to 0.39 | 0.461 |
| Pair total CYP3A5 alleles | 0 (ref) | 71 | — | 1.00 | Reference | — | — |
|  | 1 allele | 22 | –0.31 | 0.73 | –27% | –0.54 to –0.08 | 0.004 |
|  | 2 alleles | 6 | –0.60 | 0.55 | –45% | –1.00 to –0.19 | 0.002 |
| Pair total CYP3A4 alleles | 3 (ref) | 12 | — | 1.00 | Reference | — | — |
|  | 4 alleles | 87 | –0.18 | 0.84 | –16% | –0.47 to 0.11 | 0.113 |

Linear mixed-effects models accounting for 1,271 observations across 99 donor-recipient pairs. **Abbreviations:** CI, confidence interval; exp(β), exponentiated coefficient; ref, reference category. **Notes:** These models evaluate the secondary genetic determinants of tacrolimus exposure. The β coefficients represent the change in log-CDR relative to the stated reference genotype.

**Supplementary Table 3. Longitudinal linear mixed-effects regression of log-CDR stratified by combined CYP3A functional allele count**

| **Clinical phase** | **β coefficient** | **exp(β)** | **Relative change** | **95% CI (β)** | **p-value** |
| --- | --- | --- | --- | --- | --- |
| **3 total functional alleles (n = 114)** | | | | | |
| Home (ref) | — | 1.00 | Reference | — | — |
| Digestive ward | –0.92 | 0.40 | –60% | –1.26 to –0.59 | <0.001 |
| ICU | –1.06 | 0.35 | –65% | –1.39 to –0.72 | <0.001 |
| **4 total functional alleles (n = 843)** | | | | | |
| Home (ref) | — | 1.00 | Reference | — | — |
| Digestive ward | –0.64 | 0.53 | –47% | –0.75 to –0.53 | <0.001 |
| ICU | –1.09 | 0.34 | –66% | –1.20 to –0.98 | <0.001 |
| **5 total functional alleles (n = 254)** | | | | | |
| Home (ref) | — | 1.00 | Reference | — | — |
| Digestive ward | –0.53 | 0.59 | –41% | –0.70 to –0.36 | <0.001 |
| ICU | –0.78 | 0.46 | –54% | –0.96 to –0.60 | <0.001 |
| **6 total functional alleles (n = 60)** | | | | | |
| Home (ref) | — | 1.00 | Reference | — | — |
| Digestive ward | –0.52 | 0.59 | –41% | –0.81 to –0.24 | <0.001 |
| ICU | –1.05 | 0.35 | –65% | –1.37 to –0.74 | <0.001 |

Longitudinal regression models assessing changes in log-CDR across clinical phases (home, digestive ward, ICU) stratified by total combined *CYP3A* functional allele count (n = 3, 4, 5, and 6 alleles). The estimates are the β coefficients relative to the home phase (reference). Separate models were fitted for each allele stratum.
